# Macrophage-mediated chronic lymphocytic leukemia cell survival is independent of APRIL signaling

**DOI:** 10.1038/cddiscovery.2016.20

**Published:** 2016-03-21

**Authors:** MHA van Attekum, S Terpstra, E Reinen, AP Kater, E Eldering

**Affiliations:** 1 Academic Medical Center, Department of Hematology, University of Amsterdam, Meibergdreef 9, 1105 AZ, Amsterdam, The Netherlands; 2 Academic Medical Center, Department of Experimental Immunology, University of Amsterdam, Meibergdreef 9, 1105 AZ, Amsterdam, The Netherlands; 3 Lymphoma and Myeloma Center Amsterdam (LYMMCARE), Meibergdreef 9, 1105 AZ, Amsterdam, The Netherlands

## Abstract

Survival of chronic lymphocytic leukemia (CLL) cells is mainly driven by interactions within the lymph node (LN) microenvironment with bystander cells such as T cells or cells from the monocytic lineage. Although the survival effect by T cells is largely governed by the TNFR ligand family member CD40L, the exact mechanism of monocyte-derived cell-induced survival is not known. An important role has been attributed to the TNFR ligand, a proliferation-inducing ligand (APRIL), although the exact mechanism remained unclear. Since we detected that APRIL was expressed by CD68+ cells in CLL LN, we addressed its relevance in various aspects of CLL biology, using a novel APRIL overexpressing co-culture system, recombinant APRIL, and APRIL reporter cells. Unexpectedly, we found, that in these various systems, APRIL had no effect on survival of CLL cells, and activation of NF-*κ*B was not enhanced on APRIL stimulation. Moreover, APRIL stity mulation did not affect CLL proliferation, neither as single stimulus nor in combination with known CLL proliferation stimuli. Furthermore, the survival effect conveyed by macrophages to CLL cells was not affected by transmembrane activator and CAML interactor-Fc, an APRIL decoy receptor. We conclude that the direct role ascribed to APRIL in CLL cell survival might be overestimated due to application of supraphysiological levels of recombinant APRIL.

## Introduction

Interactions of chronic lymphocytic leukemia (CLL) cells with bystander cells in tumor microenvironments, such as the lymph node (LN), provide them with essential survival signals. Upregulation of pro-survival B-Cell Lymphoma-2 family members occurs on stimulation with T cells or with monocyte-derived cells such as macrophages or nurse-like cells (NLCs).^1^ Although the tumor necrosis factor (TNF) receptor ligand family member CD40L can account largely for the survival effect by T cells,^2^ several factors have been described to have a role in the CLL cell survival effect governed by monocyte-derived cells.^3,4^ A prominent factor in this context is the TNF family member a proliferation-inducing ligand (APRIL).^4^

Under physiological conditions, APRIL has diverse roles in the development of B cells. It binds to its cognate receptors transmembrane activator and CAML interactor (TACI) and B-cell maturation antigen (BCMA) after which TNF receptor associated factors are recruited to the receptor that relay the signal intracellularly. APRIL has furthermore been shown to signal via binding to heparan sulfate proteoglycans on the cell surface of its target cell.^5^ In healthy B cells, APRIL signaling has a role in the induction of CD40L-independent class-switch recombination,^6^ proliferation,^7^ and sustained survival of plasmablasts.^8^ APRIL has been reported to be expressed by several cell types including macrophages,^9^ stromal cells,^10^ CLL cells,^11^ and NLCs,^12^ which are CLL cell-differentiated monocytes that have been shown to induce survival of CLL cells.^3^ APRIL is produced as either a membrane bound or soluble factor, depending on which alternative transcript(s) is/are expressed by the cell.^13^ Furthermore, APRIL can be synthesized as part of a hybrid transcript called TWEPRIL (TWEAK-APRIL) together with TNF-related weak inducer of apoptosis (TWEAK), after which it is anchored to the cell membrane by virtue of the TWEAK domain.^14^ Both TWEPRIL and the secreted alpha transcript variant of APRIL can be cleaved by furin in the Golgi apparatus or at the cell membrane, respectively.^15^ In contrast, the membrane-bound delta variant lacks the furin cleavage domain.^13^

In its ability to support cells, APRIL contributes to the growth of several malignancies,^16^ and serum APRIL levels are correlated with worse prognosis,^17^ which was also shown for CLL.^18^ Furthermore, APRIL overexpression by transgenesis in the T-cell leukemia/lymphoma 1A (Tcl-1) CLL mouse model is associated with enhanced disease severity^19^ and APRIL transgenic mice show an enhanced proliferation of peritoneal B-1 cells,^20^ which are considered to be the precursor cells for CLL in mice.^21^ These effects are thought to result from induction of CLL cell survival by APRIL via activation of nuclear factor kappa-light-chain-enhancer of activated B cells (NF-*κ*B).^4,12^ Altogether, these data suggest a role for APRIL in CLL biology. These findings have, however, been questioned by other reports, in which no survival effect on CLL cells was found.^22,23^

To mechanistically dissect the role of APRIL, we used several complementary approaches to study its effects on CLL survival, activation, proliferation, and to investigate its role in macrophage-mediated survival. Surprisingly, we could not detect a direct effect of APRIL on CLL cells. Furthermore, although macrophages induce CLL survival, this effect appears to be independent of APRIL.

## Results

### APRIL is expressed by macrophages in the CLL LN and CLL cells express APRIL receptors

**Figure 1:**
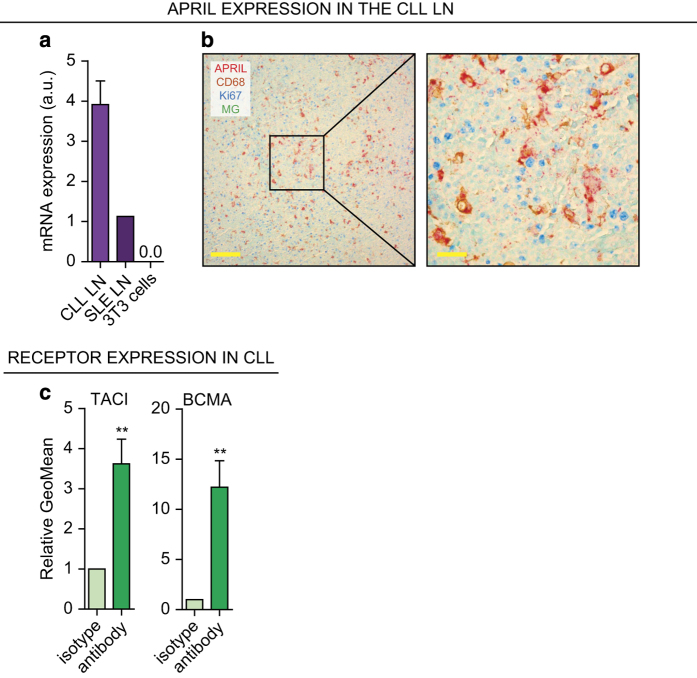
APRIL is present in the CLL LN and CLL cells express APRIL receptors. (**a**) After total RNA lysis of paraffin-embedded LN material or control NIH-3T3 (3T3) cells, APRIL mRNA levels were determined by performing a qPCR on CLL LN material (*N*=3) and an SLE LN as positive and 3T3 cells as negative control. All qPCRs were performed in triplo. a.u. denotes arbitrary units. (**b**) Paraffin embedded LN slides from six CLL patients were immunohistochemically stained for APRIL, macrophage marker CD68, proliferation marker Ki67, and nuclear counterstain methyl green (MG). Data shown are representative of *N*=6. Scale bar represents 200 *μ*m (left) or 50 *μ*m (right). (**c**) CLL cells (*N*=6) isolated from PB were stained for APRIL receptors TACI and BCMA or with the relevant isotype controls and analyzed by flow cytometry. Bars show mean±S.E.M. **P<0.01 in a paired *t*-test.

We first addressed whether APRIL is expressed in the CLL LN by performing qPCR on total RNA lysates from CLL LNs. These results show that APRIL expression in CLL LNs was ~4 times higher compared with a control systemic lupus erythematosus (SLE) LN extract. As negative control, NIH-3T3 mouse embryofibroblasts (3T3) had no APRIL expression (Figure 1a).

Next, we verified this finding on protein level using immunohistochemistry by staining for APRIL and macrophage marker CD68. As APRIL has been described to induce cell proliferation,^7^ we also stained for proliferation marker Ki67. APRIL was expressed by the large majority of CD68+ cells in both CLL and SLE LNs, but there was no spatial association with Ki67+ lymphocytes in the CLL LNs (CLL LN, Figure 1b and SLE LN, Supplementary Figure S1). Furthermore, expression of APRIL receptors BCMA and TACI was clearly detectable on CLL cells isolated from peripheral blood (PB; Figure 1c).

In summary, APRIL is expressed in the CLL LN by macrophages and APRIL receptors are present on CLL cells.

### No survival effect on CLL cells by *in vitro* APRIL stimulation

**Figure 2:**
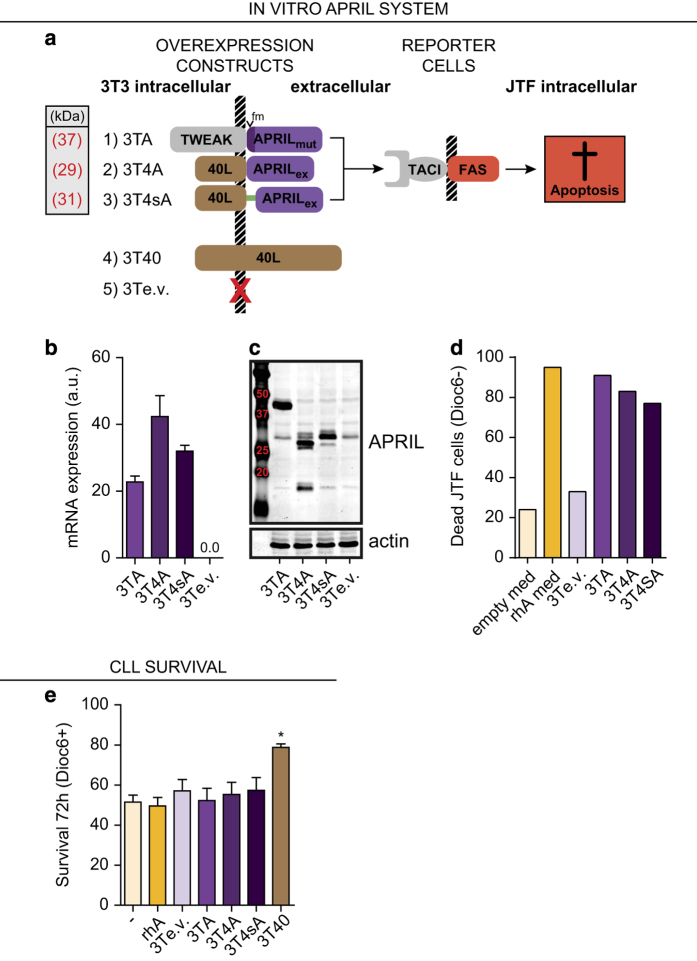
APRIL does not induce CLL cell survival. (**a**) Depiction of APRIL overexpressing cell lines, control cell lines, and reporter cells used in co-culture experiments. NIH-3T3 cell lines overexpressing three different membrane-bound APRIL constructs were created (Methods section). Apoptosis in APRIL reporter JTF cells is induced on APRIL signaling, as TACI signaling triggers the FAS cell-death pathway. Full-length CD40L overexpressing 3T3 cells (3T40) and empty-vector transduced 3T3 cells (3Te.v.) are used as controls. Mutated furin sites are indicated by ‘fm’, the spacer region is depicted by a green line. All constructs are drawn to scale. (**b**) APRIL mRNA expression levels of the different APRIL overexpressing cell lines were tested by qPCR and compared with cells overexpressing 3Te.v. The qPCR was performed in triplo and bars show mean±S.E.M., a.u. denotes arbitrary units. (**c**) APRIL protein expression levels of the different APRIL overexpressing cell lines were tested by western blot and compared with cells overexpressing 3Te.v. The predicted molecular weights of the APRIL fusion proteins are indicated in Figure 2a. (**d**) Cell lines described in Figure 2a were seeded as feeder layers, and JTF reporter cells^27^ were plated on top. Concurrently, JTF reporter cells were cultured in conditioned medium from APRIL (rhA med) or mock (empty med) transfected HEK293T cells. After 24 h co-culture, the percentage of dead (Dioc6 negative) JTF reporter cells was determined by Dioc6-PI staining. (**e**) CLL cells were cultured for 72 h without stimulation (–) or with 200 ng/ml rhA. Likewise, CLL cells were co-cultured on the indicated APRIL expressing or control cell lines. Next, survival was determined by Dioc6-PI staining. Viable cells were defined as Dioc6-positive cells. CD40L overexpressing feeder cells (3T40) were used as a positive control for CLL cell survival. Bars show mean±S.E.M. for *N*⩾8 for each condition. *P<0.05 in a ANOVA with Tukey *post hoc* tests. When testing for significant differences, rhA was compared with unstimulated cells and 3T3 overexpression cell lines to 3Te.v.

To explore direct functional effects of APRIL on CLL cells, we transduced NIH-3T3 cells (DSMZ, Braunschweig, Germany) with three different membrane-docked APRIL constructs (Figure 2a). We thus generated a system similar to the widely used TNF family member CD40L overexpressing NIH-3T3 line (3T40),^24–26^ thereby ensuring trimerization of APRIL and expression on the cell membrane. The first cell-line expresses the membrane-bound TWEPRIL hybrid mRNA, with mutated furin consensus sites to render it uncleavable (3TA). In the second and third constructs (3T4A and 3T4sA), the intracellular and transmembrane regions of CD40L were fused to the extracellular domain of APRIL, without or with an interposed spacer (‘s’) region. The 3T40 cell line^24–26^ was used as a control.

APRIL expression in these cell lines was then verified by qPCR (Figure 2b) and western blot (Figure 2c), and signaling competence was tested using Jurkat-TACI:FAS (JTF) reporter cells^27^ (Figure 2d). These JTF cells undergo apoptosis on TACI signaling as a result of intracellular FAS domains, and provide a sensitive read-out for APRIL binding to its cognate receptor (Figure 2a). Conditioned medium from APRIL overexpressing HEK293T cells (rhA med) and recombinant human APRIL (data not shown) were included as controls (Figure 2d). These data showed that all cell lines from our *in vitro* co-culture system express APRIL and that the expressed APRIL is able to signal via TACI.

These APRIL expressing 3T3 cells were subsequently used to test whether APRIL induced CLL cell survival. In contrast to 3T40 cells, we found no survival effect by any of the APRIL constructs or by rhA after 72 h co-culture (Figure 2e). Similarly, we could not detect a survival effect of conditioned supernatant from APRIL transfected HEK293T cells compared with supernatant from mock transfected cells (data not shown and Supplementary Figure S2). Using the same APRIL stimuli, survival of CLL cells was measured at later time points (3, 6 and 10 days). In accordance with the results obtained at *T*=72 h, APRIL did not significantly increase CLL survival, although a minor effect could be observed at day 10 for rhA (Supplementary Figure S2), as reported before.^28^

### No NF-*κ*B activation, activation marker expression, or cell division in CLL cells exposed to APRIL

**Figure 3:**
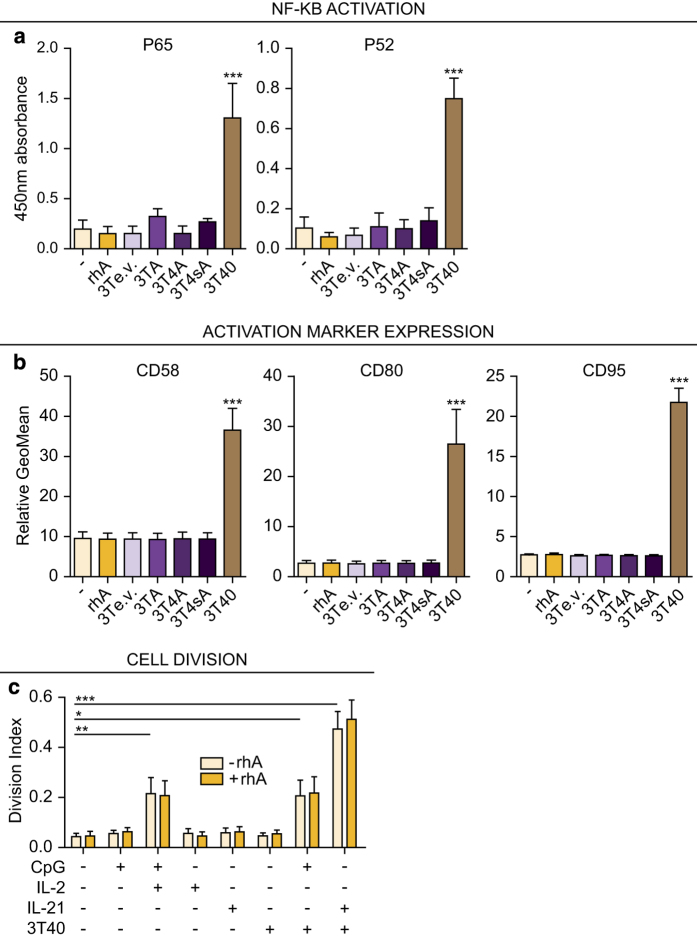
APRIL does not induce NF-*κ*B signaling, activation marker expression, or cell division in CLL cells. (**a**) CLL cells were cultured as in Figure 2e and nuclear extracts were prepared after 24 h. The binding of activated canonical p65 and non-canonical p52 NF-*κ*B subunits to consensus sequence oligonucleotides was then determined using enzyme-linked immunosorbent assay (ELISA). CD40L overexpressing feeder cells (3T40) were used as a positive control for NF-*κ*B activation. Bars show mean±S.E.M. for *N*=3 for 3TA and 3T4sA and *N*=5 for the other conditions, respectively. ***P<0.001 in an ANOVA test with Tukey *post hoc* analysis. When testing for significant differences, rhA was compared with unstimulated cells and 3T3 overexpression cell lines to empty-vector transduced 3T3 cells (3Te.v.). (**b**) CLL cells were cultured as in Figure 2e for 72 h and expression levels of activation markers CD58, CD80 and of CD95 were determined using flow cytometry. CD40L overexpressing feeder cells (3T40) were used as a positive control for activation marker induction. Bars show mean±S.E.M. for *N*=3. ***P<0.001 in an ANOVA test for repeated measures with Tukey *post hoc* analysis. When testing for significant differences, rhA was compared with unstimulated cells and 3T3 overexpression cell lines to 3Te.v. (**c**) CFSE-stained CLL cells were cultured with various stimulations as indicated and as described,^2^ each time with or without rhA. After 4 days, the CFSE dilution was visualized by flow cytometry and division indices were calculated. Bars show mean±S.E.M. for *N*=6. *P<0.05; **P<0.01; ***P<0.001; Each stimulation without rhA (light colored bars) was compared with the unstimulated condition in an ANOVA test for repeated measures with Dunnet’s *post hoc* analysis and the differences between − and +rhA for each condition were determined using a two-way ANOVA with Bonferroni correction.

As several TNF family members can induce NF-*κ*B,^29^ we investigated if APRIL is able to induce NF-*κ*B activation by performing an NF-*κ*B DNA-binding enzyme-linked immunosorbent assay and found that, as expected, 3T40 cells induced both the canonical (p65) and non-canonical (p52) pathway in CLL cells. In contrast, no NF-*κ*B activation could be detected after stimulation with various APRIL constructs or rhA (Figure 3a), and known NF-ĸB target transcripts were not induced (data not shown).

Strong CD40 stimulation via cell-bound CD40L induces high-level NF-*κ*B activation in CLL cells. We have previously found that a CD40 stimulating antibody that induces moderate stimulation is able to upregulate activation markers CD58, CD80 and also CD95 (data not shown), indicating a higher sensitivity of this read-out. We therefore tested the potential of APRIL in this context, but in contrast to CD40L stimulation, APRIL stimulations did not upregulate the indicated markers (Figure 3b).

To study APRIL’s potential involvement in CLL cell proliferation,^16^ cell division was traced using carboxyfluorescein succinimidyl ester (CFSE) labeling, and the division index was calculated after various proliferation stimuli in the presence or absence of rhA. In line with a previous report,^2^ we found an increased proliferation of CLL cells after stimulation with CpG+interleukin (IL)2, 3T40+CpG, and 3T40+IL21, but no effect of rhA either as a single agent or in combination with these stimuli (Figure 3c).

Summarizing, although we found that CD40L is able to induce NF-*κ*B activation in CLL cells, activation marker expression and cell proliferation in combination with CpG or IL21, similar effects were not detectable after APRIL stimulation.

### Macrophage-mediated CLL survival is independent of APRIL

We^30^ and others^3^ have previously found that monocyte-derived cells such as macrophages are able to induce survival of CLL cells, and it was suggested that survival by monocyte-derived cells is dependent on APRIL.^12^ Although we did not observe a survival effect of stimulation with APRIL as a single stimulus (Figure 2e), the effects of APRIL could be dependent on other macrophage-expressed cytokines.

**Figure 4:**
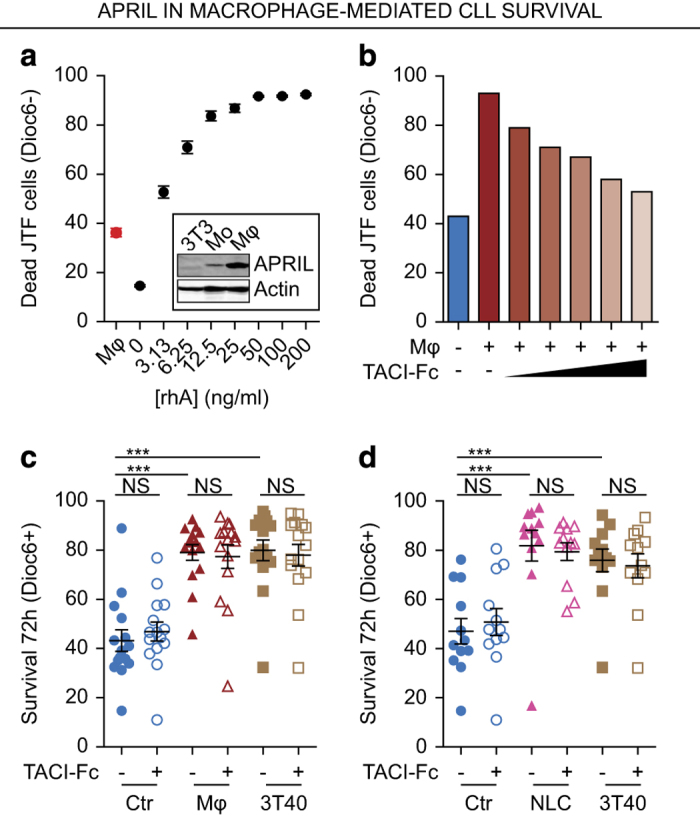
APRIL is expressed by macrophages, but has no role in macrophage-mediated survival. (**a**) JTF reporter cells were stimulated for 24 h with different concentrations of rhA or with M1-differentiated macrophages. Consequently, cell viability was determined as in Figure 2d and the macrophage-induced cell death was plotted alongside of the rhA titration curve. All conditions were performed in triplo and mean±S.E.M. are shown. Inset: APRIL expression was determined in these macrophages (Mφ) by western blot and compared with monocytes (Mo) and untransduced 3T3 cells as negative control. (**b**) JTF reporter cells were stimulated with M1-differentiated macrophages as in Figure 4a in the presence of increasing concentration of the APRIL decoy receptor TACI-Fc (from 0.25 *μ*g/ml to 2.5 *μ*g/ml) or control IgG after which cell viability of the JTF cells was measured as in Figure 2d. (**c**) Confluent feeder layers of macrophages (Mφ) were generated as in Figure 4a and 3T40 feeder layers as in Figure 2e. These feeder layers or empty wells (Ctr) were then pre-incubated for 30 min with TACI-Fc to suppress APRIL signaling or control IgG after which CLL cells were added on these feeder layers and co-cultured for 72 h. Next, survival of the CLL cells was determined as in Figure 2e. Each point is one CLL sample (*N*=15) cultured in the indicated condition and mean±S.E.M. are indicated. ***P<0.001; NS, not significant, in an ANOVA test for repeated measures with Dunnett’s *post hoc* analysis. (**d**) Confluent feeder layers of NLCs were generated by differentiating monocytes for 10 days using CLL cells. After washing, their survival effect on CLL cells in the presence of absence of TACI-Fc was determined as in Figure 4c. Each point is one CLL sample (*N*=12) cultured in the indicated condition and mean±S.E.M. are indicated. ***P<0.001; NS, not significant, in an ANOVA test for repeated measures with Dunnett’s *post hoc* analysis.

We, therefore, first generated M1 macrophages *in vitro* by differentiating healthy donor-isolated monocytes with interferon gamma (IFN-Y; R&D systems, Minneapolis, MN, USA). We then tested whether APRIL was expressed by these macrophages on western blot and found high expression in differentiated macrophages compared with low expression in monocytes and no expression in control 3T3 cells (Figure 4a inset and Supplementary Figure S3). The APRIL signaling capacity of these macrophages was then tested by comparing cell-death induced by macrophages in JTF reporter cells with the JTF death-to-rhA dose-response curve. The APRIL signaling capacity of macrophages was between that of 0 and 3.13 ng/ml rhA (Figure 4a).

To inhibit potential APRIL signaling during macrophage stimulation, we used TACI-Fc (R&D systems), a chimeric decoy receptor for APRIL.^31^ We tested the activity of TACI-Fc by its ability to inhibit macrophage-induced cell death of JTF reporter cells cultured on macrophages. We found that TACI-Fc dose-dependently reduced APRIL signaling from macrophages (Figure 4b).

We then cultured CLL cells on macrophages and measured CLL survival in the absence or presence of 2.5 *μ*g/ml TACI-Fc, the concentration at which macrophage-induced APRIL signaling was completely reverted. These data show that abrogation of APRIL signaling did not decrease the survival effect conveyed by macrophages (Figure 4c), suggesting no direct role for APRIL in macrophage-mediated CLL survival. Similarly, when culturing CLL cells on NLCs^12^ generated by 10 days stimulation of monocytes with CLL cells, inhibition of APRIL signaling by TACI-Fc did not affect CLL survival (Figure 4d).

## Discussion

We studied potential effects of TNF-family member APRIL on CLL cells, using complementary approaches and JTF reporter cells to verify the functionality of recombinantly expressed APRIL and the TACI-Fc decoy receptor. In contrast to our initial expectations, we could not detect an effect of APRIL on either CLL cell survival, cell activation, NF-*κ*B activation or cell proliferation. In addition, we could not detect a direct role of APRIL in macrophage-mediated CLL cell survival.

Various studies reported on the effects of APRIL on CLL. Although some publications show an increased *in vitro* survival of CLL cells by rhA when used at a concentration of 500 ng/ml,^4,12^ our experiments using 200 ng/ml rhA (Figure 2e) are in line with the data of several other groups that were unable to find effects of recombinant APRIL, either alone^22^ or in combination with B-cell activating factor and chemokine (C-X-C motif) ligand 1 (CXCL)12.^23^ Also, we established that the amount of APRIL produced by macrophages is >100 orders of magnitude lower compared with concentrations used in the reports that detect survival by APRIL. Although APRIL may induce survival at high concentrations,^4,12^ this effect might be supraphysiological. Furthermore, concerning the survival effect of APRIL on non-malignant B cells, several groups have shown that APRIL is also dispensable in this context.^32^

In the Tcl-1 mouse model for CLL, we found that overexpression of human APRIL results in enhanced disease progression and shorter survival.^19^ In light of these results, our current *in vitro* findings were also unexpected. In the APRIL overexpressing Tcl-1 model, the construct encoding human APRIL is under control of the Lck promoter. APRIL is thus predominantly expressed by T cells, and is present in the serum at a concentration comparable to our *in vitro* systems (data not shown). As T cells not only interact with other lymphoid cells including (leukemic) B cells but also with myeloid-derived immune cells, it cannot be ruled out that the observed effects occur indirectly via other cells in the tumor microenvironment. Theoretically, the differences could also be due to distinct APRIL effects in the mouse compared with human situation.

We found no evidence that CLL proliferation is enhanced either *in vitro* or *ex vivo* by APRIL. These data are in line with another publication in which no significant proliferative effect of APRIL medium in the presence of CpG was found.^28^ Studies on the effects of APRIL on proliferation of healthy B cells have been inconclusive; APRIL knockout mice for instance show normal B-cell proliferation *in vitro* ^33^ and mice deficient for the APRIL receptor TACI paradoxically show increased B-cell proliferation,^34^ whereas BCMA knockout mice show no overt phenotype.^35^

In conclusion, our data indicate that APRIL does not directly mediate survival and proliferation of CLL cells. Consequently, APRIL signaling as therapeutic target in CLL might be beneficial in consideration that potential effects might be indirect.

## Materials and methods

### Patient samples

Patient material was obtained from CLL patients, after written informed consent and approval by our Ethical Review Board in agreement with the Helsinki Declaration of 1975, revised in 1983, as described before.^36^ All samples contained at least 90% CD5^+^/CD19^+^ cells (Supplementary Table S1). In all experiments, CLL cells were used at a final concentration of 1.5x10^6^ cells/ml.

### APRIL overexpression cell lines and other APRIL stimulations

Mouse embryofibroblasts NIH-3T3 cells were transduced for stable overexpression with pBABE vectors expressing (1) TWEPRIL (NM_172089.3) with mutated furin cleavage sites (92RR→AA and 104RR→AA), (2) the transmembrane domain of CD40L (amino acids 1–112 of NM_000074.2) fused to the extracellular part of APRIL (amino acids 105-252 of NM_003808.3) without a linker region, or (3) with a linker region (PAAAAAASAAAAAAWVPVAT; Figure 2a), (4) CD40L,^37^ or (5) empty vector, and all transduced cells were selected using puromycin. All constructs were sequence verified before transduction. Cells were cultured in IMDM supplemented with (IMDM+/+): 10% fetal bovine serum (FBS, Invitrogen, Carlsbad, CA, USA), 100 u/ml Penicillin-100 *μ*g/ml Streptomycin (Life Technologies, Austin, TX, USA), 2  mM L-glutamine (Life Technologies) and 0.00036% *β*-mercaptoethanol (Sigma, St. Louis, MO, USA). When used as adherent feeder layer, fibroblasts were irradiated (30 Gy) to stop proliferation before being seeded. After feeder cell adhering, CLL cells were plated on the respective cells. Where indicated (rhA) 200 ng/ml rhA was added to the culture medium, or culture medium conditioned on rhA-overexpressing HEK293T cells was added to the CLL cells at 80% final volume (rhA med).

### Immunohistochemistry

Paraffin embedded CLL LN tissue was obtained from our institute’s pathology department. Four-micron sections were de-waxed by immersion in xylene and hydrated by serial immersion in ethanol and TBS. Antigen retrieval was performed by heating sections for 20 min in sodium citrate buffer (10 mM sodium citrate, 0.05% Tween20, pH 6.0). Sections were washed with TBS (2×10 min) and blocked with Ultra V block (Thermo Scientific, Waltham, MA, USA) for 10 min at T_room_. Sections were incubated with primary antibody APRIL-y2 (1:1000 Enzo LifeSciences, Farmingdale, NY, USA) in normal antibody diluent (ImmunoLogic, Duiven, The Netherlands) overnight at 4 °C. After washing, sections were incubated with post-antibody block (ImmunoLogic) and subsequently incubated with secondary Polymer a-Rb/AP antibody (ImmunoLogic) followed by visualization by Vector Red (Vector Laboratories, Burlingame, CA, USA). A second antigen retrieval was performed for 10 min at 98 °C in TRIS-EDTA (pH=9.0) and after Ultra V block (Thermo Scientific), sections were incubated with a combination of primary antibodies directed against CD68 (PG-M1, Dako, Carpinteria, CA) and Ki67 (SP6, Klinipath, Duiven, The Netherlands), both 1:2000 in normal antibody diluent for 1 h at T_room_. After washing, a combination of Polymer a-Rb/AP and Polymer a-Ms/HRP (both Immunologic) was added for 30 min at T_room_ and antibody binding was visualized by Vector Blue (Vector Laboratories) and subsequent DAB (ImmunoLogic) staining after which slides were counterstained with methyl green and mounted with vectamount. Slides were visualized using a Leica DMLB microscope (Leica Microsystems, Buffalo Grove, IL, USA) equipped with a Leica DFC420 camera and cropped using Adobe Illustrator CS5 software (Adobe, San Jose, CA, USA).

### APRIL reporter cell assays

To measure APRIL signaling, JTF reporter cells, provided as a kind gift by P. Schneider,^27^ were cultured with different APRIL stimuli for 24 h, after which cell death of JTF cells was measured by Dioc6-PI staining as described before.^36^

### Flow cytometry and cell viability

Cell viability was measured by Dioc6-PI staining as described before.^36^ Flow cytometrical staining for APRIL receptors was performed using the TACI-PE (BD Bioscience, San Jose, CA, USA) and BCMA-FITC (Enzo) antibodies as described previously^2^ and stained cells were analyzed on a FACS Canto II (BD). Data was then analyzed using FlowJo 9.9 (FlowJo LLC, Ashland, OR, USA).

### Western blot

Western blotting was performed as described previously,^38^ using the *α*-human APRIL-y1 antibody (Abcam, Cambridge, MA, USA) and *β*-actin (Santa Cruz, Dallas, TX, USA) as a loading control. IRDye 680 donkey anti-rabbit IgG and IRDye 800 donkey anti-goat IgG (Westburg, Leusden, The Netherlands) were used as secondary antibodies.

### Real-time polymerase chain reaction (qPCR)

Total RNA was isolated from paraffin-embedded CLL LN material or from APRIL overexpressing 3T3 cells using the GeneElute Mammalian Total RNA Miniprep kit (Sigma) and cDNA was created by reverse transcriptase reaction according to manufacturer’s instructions (Promega, Madison, WI, USA). APRIL and household gene hypoxanthine phosphoribosyltransferase 1 (HPRT) were amplified using exon–exon boundary overlapping probes (APRIL 5ʹ-CTGCTATAGCGCAGGTGTCTT-3ʹ and 5ʹ-GGAAGGTTCCATGTGGAGAG-3ʹ; HPRT 5ʹ-CCTGGCGTCGTGATTAGTGA-3ʹ and 5ʹ-CGAGCAAGACGTTCAGTCCT-3ʹ) in a SYBR green (Life Technologies, Austin, TX, USA) reaction (40 cycles of 3 s at 95 °C followed by 30 s at 60 °C). The expression of APRIL was then calculated per sample as the difference in Ct values between the APRIL signal and HPRT signal using the formula 1000x2^-(Ct APRIL - Ct HPRT)^.

### Cell proliferation assays

Cell proliferation was assessed by CFSE cell tracing as described before,^2^ using 200 ng/ml rhAPRIL (rhA; Peprotech, Rocky Hill, NJ, USA) and other reagents as described before.^2^ Division indices were calculated using FlowJo 9.9 (FlowJo LLC).

### Macrophage and NLC experiments

Monocyte-derived macrophages and NLCs were obtained by differentiating monocytes isolated from healthy donor buffy coats obtained from the central blood bank after obtaining written informed consent. To this end, PBMCs were isolated using ficoll gradient purification (Lucron, Dieren, The Netherlands), after which monocytes were separated from PB lymphocytes using percoll gradient purification (GE healthcare, Milwaukee, WI, USA), both according to manufacturer’s instructions. Next, monocytes were incubated to adhere at 37 °C in 5% CO_2_ for 40 min at a concentration of 0.75×10^6^ cells/ml in 6-well plates (3 ml) in IMDM/1% FBS and washed to remove non-adherent cells. The monocytes were then differentiated towards M1 macrophages using 10ng/ml IFN-Y in IMDM+/+ for 72 h or to NLCs by differentiating them using CLL cells for 10 days.^3^ After washing the macrophages or NLCs twice, they were pre-incubated for 30 min with TACI-Fc or an equimolar concentration of control IgG (R&D systems) to obtain a final concentration of 2.5 *μ*g/ml of TACI-Fc. Next, thawed CLL cells were added. After 72 h, cell viability was measured as described before.^36^

### Statistical analysis

One-way analysis of variance (ANOVA) with Tukey *post hoc* tests (comparing all groups to each other) or Dunnet’s *post hoc* test (comparing all groups to one group) were performed to test for significant differences between multiple groups. When applicable, tests were adjusted for repeated measures. A two-way ANOVA with Bonferroni *post hoc* tests was used when testing for differences between groups with two independent variables. When testing for differences between two groups, a *t*-test was used. *P*-values<0.05 (*), *P*<0.01 (**), and *P*<0.001 (***) were considered statistically significant, non-significance is not indicated except in Figures 4c and d.

## Abbreviations

APRIL: a proliferation-inducing ligand
BCMA: B-cell maturation antigen
CLL: chronic lymphocytic leukemia
IFN-Y: interferon gamma
IL: interleukin
JTF: Jurkat-TACI:FAS (reporter cells)
LN: lymph node
NF-*κ*B: nuclear factor kappa-light-chain-enhancer of activated B cells
PB: peripheral blood
rhA: recombinant human APRIL
SLE: systemic lupus Erythematodes
TACI: transmembrane activator and CAML interactor
TNF: tumor necrosis factor
Tcl-1: T-cell leukemia/lymphoma 1A
ANOVA: one-way analysis of variance
HPRT: hypoxanthine phosphoribosyltransferase 1
CFSE: carboxyfluorescein succinimidyl ester
NLCs: nurse-like cells.
