# Supplementary figures and images for: Macrophage-mediated chronic lymphocytic leukemia cell survival is independent of APRIL signaling
Source: Cell Death Discov. 2016 Mar 21;2:16020–. doi: 10.1038/cddiscovery.2016.20 (PMC4979474; doi:10.1038/cddiscovery.2016.20)

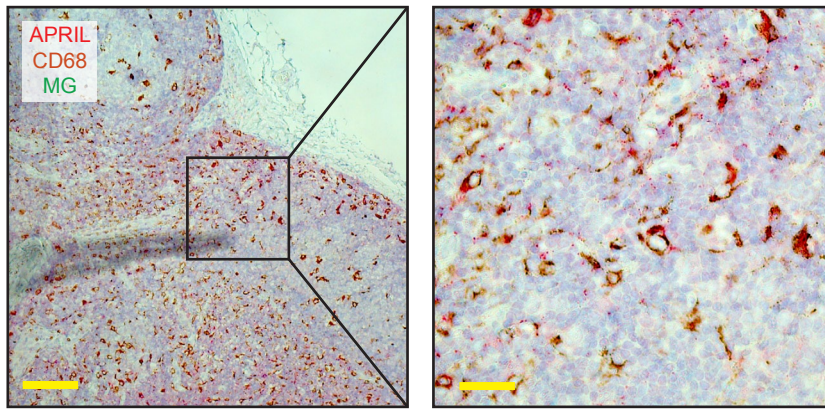

Supplement: Supplementary Figure S1 [file cddiscovery201620-s1.pdf]

No direct role for APRIL in macrophage mediated CLL survival fig S2

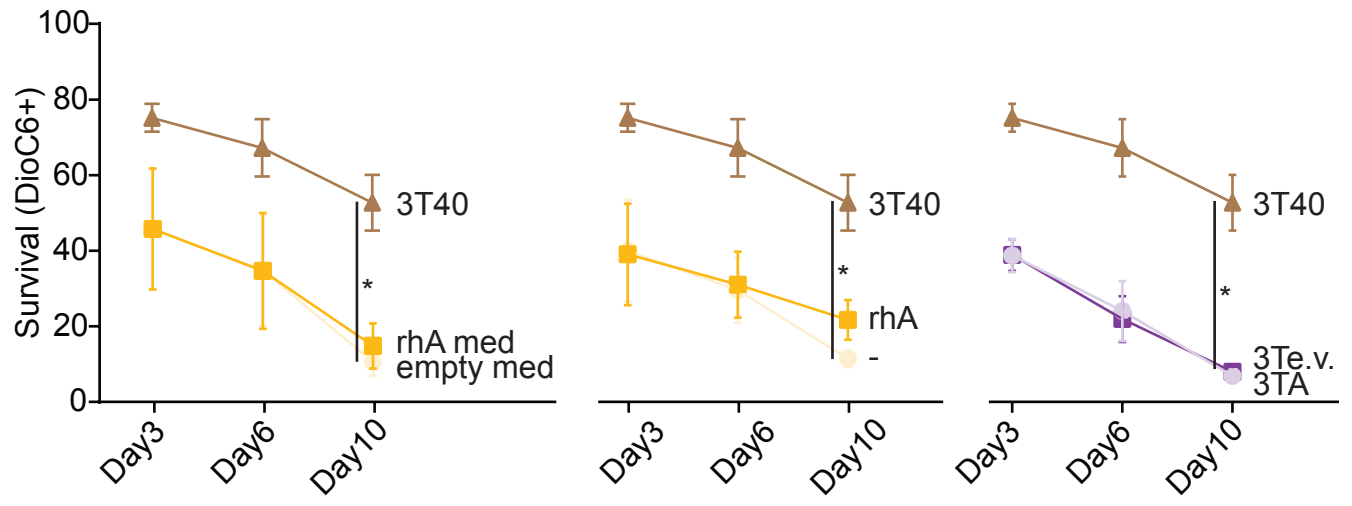

Supplement: Supplementary Figure S2 [file cddiscovery201620-s2.pdf]

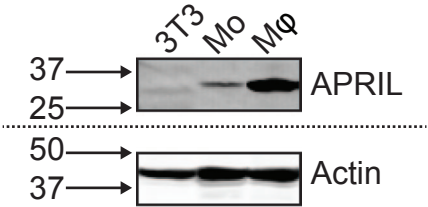

Supplement: Supplementary Figure S3 [file cddiscovery201620-s3.pdf]
