# Supplementary material for: Macrophage-mediated chronic lymphocytic leukemia cell survival is independent of APRIL signaling
Source: Cell Death Discov. 2016 Mar 21;2:16020–. doi: 10.1038/cddiscovery.2016.20 (PMC4979474; doi:10.1038/cddiscovery.2016.20)
Supplement: Supplementary Figures Legend [file cddiscovery201620-s5.doc]

Figure S1: CD68-APRIL staining in SLE LN

A paraffin embedded LN slide from an SLE patient was immunohistochemically stained for APRIL, macrophage marker CD68, and nuclear counterstain Methyl Green (MG). Scale bar represents 200μm (left) or 50μm (right).

Figure S2: Long term survival of CLL cells using different APRIL stimuli

CLL cells were cultured with the APRIL stimulations used in Figure 2d and Figure 2e and survival was measured at indicated time points. All CLL samples were pre-incubated O/N with 1.5μg/mL cytosine guanine dinucleotide to induce TACI and BCMA upregulation^28^. Points show mean ± s.e.m. for N=3 patients for each condition. *, P<0.05 in a paired T-test comparing stimulated conditions (APRIL stimulations or 3T40) with the respective control conditions (-, empty med, 3Te.v.). Only significantly different data points at day10 are indicated for readability.

Figure S3: Size markers for western blot of Figure 4a

Size markers for the western blot of Figure 4a (inset) are indicated by arrows. The predicted mass for APRIL is 27kDa.
